# Intramuscular hemangioma with hemorrhagic transformation arising from paraspinal muscles of posterior neck: A case report

**DOI:** 10.1097/MD.0000000000021741

**Published:** 2020-08-14

**Authors:** Dongwoo Yu, Joon Hyuk Choi, Ikchan Jeon

**Affiliations:** a Department of Neurosurgery; b Department of Pathology, Yeungnam University Hospital, Yeungnam University College of Medicine, Daegu, South Korea.

**Keywords:** intramuscular, hemangioma, hemorrhage, posterior neck

## Abstract

**Rationale:**

Hemangiomas are usually found in cutaneous or mucosal layers, less than 1% of hemangiomas develop in skeletal muscles. Intramuscular hemangioma (IH) in the head and neck areas is relatively infrequent, accounting for 15% of IH. Most of them are identified as a benign mass, and rapid changes in size or internal bleeding are rare.

**Patient concerns:**

A 60-year-old female patient presented with a 2-week history of sudden onset posterior neck pain. There was no neurological deficit except limited neck motion due to pain. The palpable mass was noted on the paraspinal muscles of cervicothoracic junction, which was located midline to left side portion with tenderness.

**Diagnoses:**

Magnetic resonance imaging demonstrated a round shaped, multi-lobulated, and well-defined mass lesion (4.1 × 2.6 × 0.9 cm) embedded from the inter-spinous space of T1-2 to the left paraspinal muscles. The lesion was iso-intense on T2-weighted images (WI), iso- to slightly low-intense on T1-WI, heterogeneous enhancement of intra- and peri-mass lesion on contrast-enhanced T1-WI. Vascular structures presented as signal voids were identified internally and around the mass lesion. Histological examination revealed a mixed-type hemangioma.

**Interventions:**

The mass was removed completely including some of the surrounding muscles where boundaries were unclear between the mass and surrounding muscles with ligation of peritumoral vessels. Dark-brown colored blood was drained from the ruptured tumor capsule during the dissection. There was no bony invasion.

**Outcomes:**

The preoperative symptoms improved immediately after the operation. There is no residual or recurrence lesion by the 15-months follow-up.

**Lessons:**

IH with hemorrhagic transformation in the head and neck is extremely rare. In the case of intramuscular tumors accompanied by a sudden onset of severe acute pain, we recommend considering a differential diagnosis of IH with hemorrhagic transformation. Complete resection of the tumor mass including surrounding muscles is required to prevent recurrence.

## 1 Introduction

Hemangiomas are the most common benign endothelioma in infancy, accounting for approximately 7% of soft tissue tumors and are usually found in cutaneous or mucosal layers.^[1]^ Although the cause of the tumor is not clear, it is highly probable that it is congenital, and will disappear naturally by the age of 12 years.^[2,3]^ Hemangiomas of skeletal muscles, intramuscular hemangioma (IH) account for less than 1% of the total hemangiomas and occur mainly in the trunk and limbs.^[4,5]^ Occurrence in the head and neck areas are relatively infrequent (15% of IH), but within these areas, the masseter muscle is the most commonly affected site, followed by the trapezius and temporalis muscles.^[6]^ IH are usually found as an incidental benign palpable mass, but accurate diagnosis before surgery is difficult due to non-specific symptoms, deep anatomical positions, and a low frequency of occurrence.^[4,7]^

As most IH develop congenitally and are identified as an incidental benign mass, mass growth or hemorrhagic change is uncommon. We report a rare case of IH with hemorrhagic transformation arising from the paraspinal muscles of the posterior neck, around the cervicothoracic junction.

## 2 Case presentation

Patient has provided written informed consent for publication of this case report and any accompanying images.

A 60-year-old female patient presented with a 2-week history of a sudden onset natured posterior neck pain around the cervicothoracic junction and aggravation over the last week. The patient had a history of lipoma removal on the back 2 years ago as well as hypertension and diabetes. On neurological examination, she demonstrated no definite neurological deficits except limited neck motion caused by pain. The palpable mass was noted on the area of paraspinal muscles, which was located midline to left side portion with tenderness. The mass had a fixed in nature and was 4.1 × 2.6 × 0.9 cm in size.

**Figure 1:**
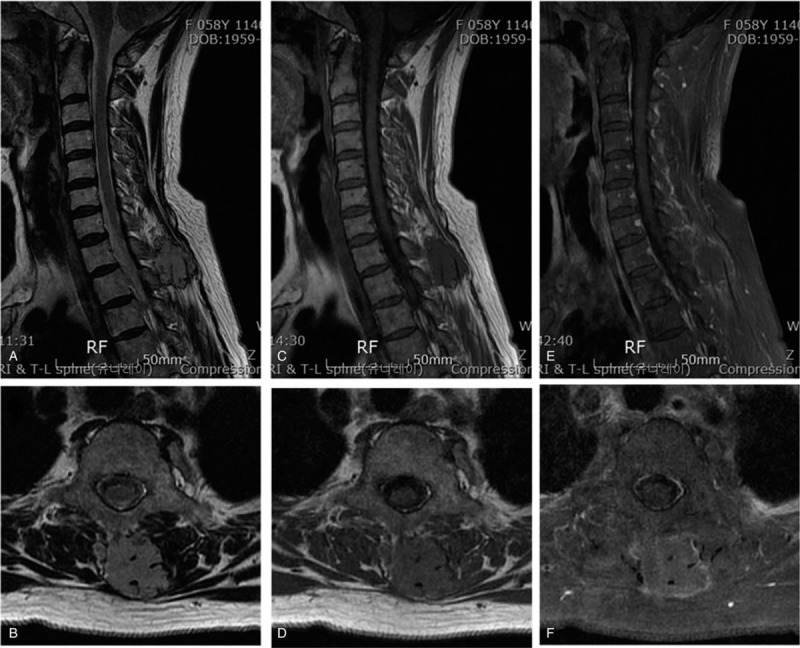
Preoperative magnetic resonance imagings demonstrated a round shaped, multi-lobulated, and well-defined mass lesion embedded from the inter-spinous space at the T1-2 level to the left paraspinal muscles. The lesion was iso-intense on T2-weighted images (WI) (A, B), iso- to slightly low-intense on T1-WI (C, D), heterogeneous enhancement of intra- and peri-mass lesion on contrast-enhanced T1-WI (E, F). Vascular structures presented as signal voids were identified internally and around the mass lesion.

Preoperative magnetic resonance imaging (MRI) demonstrated a round shaped, multi-lobulated, and well-defined mass lesion embedded from the inter-spinous space at the T1-2 level to the left paraspinal muscles. The lesion was iso-intense on T2-weighted images (WI), iso- to slightly low-intense on T1-WI, heterogeneous enhancement of intra- and peri-mass lesion on contrast-enhanced T1-WI. Vascular structures presented as signal voids were identified internally and around the mass lesion (Fig. 1).

**Figure 2:**
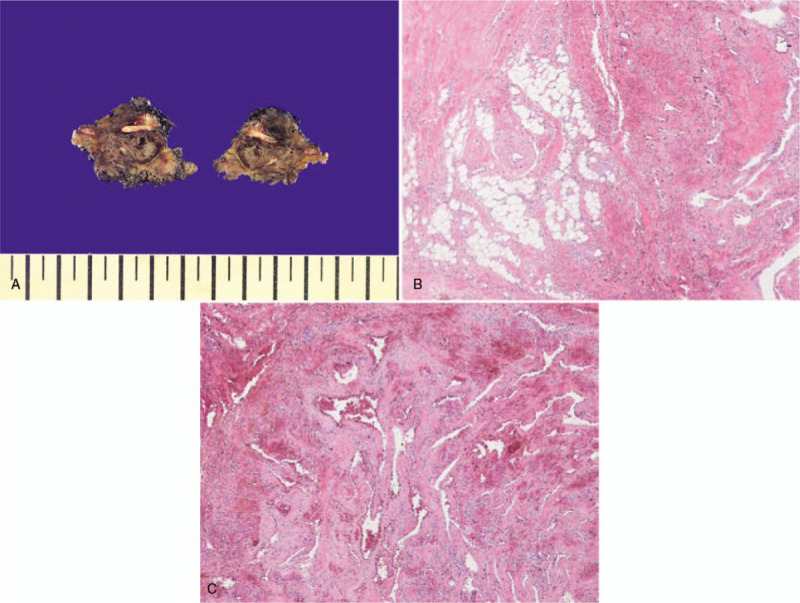
The mass was removed completely including some of the surrounding muscles where boundaries were unclear between tumor and the surrounding muscles using the en bloc method with ligation of peritumoral vessels (A). On the histological examination, variably sized vascular channels within skeletal muscles and mature adipose tissue are present (hematoxylin-eosin stain, ×40) (B). There are thin-walled vascular spaces and hemorrhage (hematoxylin-eosin stain, ×100) (C).

Surgery was performed with a vertical midline skin incision from C7 to T2. The mass was relatively well separated from the surrounding muscles. A part of tumor capsule was ruptured, and dark-brown colored blood was drained. There was no evidence of invasion to the surrounding bony structures including spinous process and lamina. The mass was removed completely including some of the surrounding muscles where boundaries were unclear between tumor and the surrounding muscles using the en bloc method with ligation of peritumoral vessels. On the histological examination, variably sized vascular channels within skeletal muscles and mature adipose tissue are present (hematoxylin-eosin stain, ×40). There are thin-walled vascular spaces and hemorrhage. (hematoxylin-eosin stain, ×100) (Fig. 2). Histological examination confirmed a mixed-type hemangioma.

**Figure 3:**
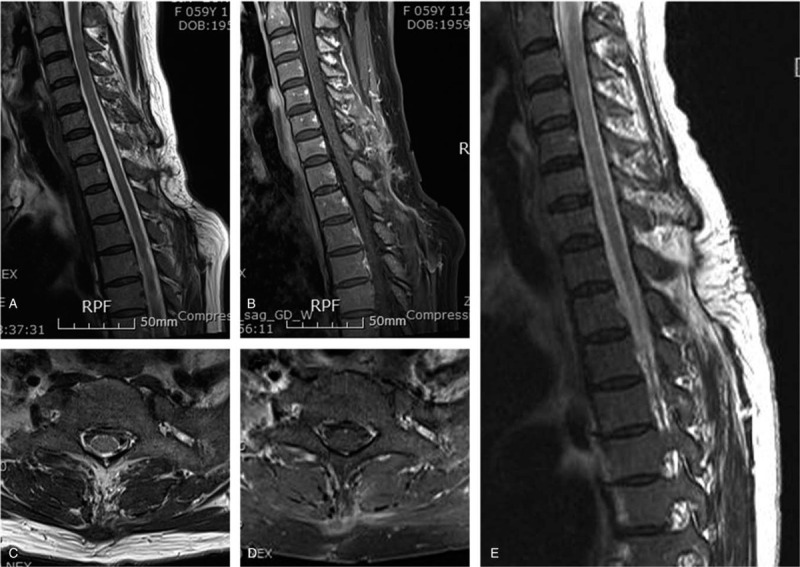
The preoperative symptoms improved immediately after the operation, and the magnetic resonance imagings conducted after 7 (A-D)- and 15 (E)-month postoperatively revealed no residual or recurrence lesion.

The preoperative symptoms improved immediately after the operation, and the MRIs conducted after 7 and 15-month postoperatively revealed no residual or recurrence lesion (Fig. 3).

## 3 Discussion

IH was first reported by Liston in 1843. IH is a non-metastatic congenital benign tumor, and approximately 50% of cases remain undetected until related symptoms are present.^[7]^ In contrast to the common type of skin hemangioma in infants, IH usually occurs between the ages of 10 and 20 years. It is generally known that IH occurs regardless of the sex.^[8]^ Clinical features of IH appear in the form of a locally developed, palpable soft mass with pain is the most common symptom. As the tumor grows, pain can be developed by the pressure of the enlarged tumor (pressure-induced pain) without an invasion of surrounding nerve components. Thrills, bruits, pulsations, and skin color changes are uncommon unless tumor has invaded large vessels.^[8]^

MRI is a useful method for providing better detection and delineation of the extent of IH. MRI findings suggestive of IH are

1) hyper-intensity on both T1- and T2-WI
2) serpiginous pattern, septated-striated high signal channels and curvilinear areas of low intensity consistent with vascular spaces
3) focal heterogenicities representing areas of thrombosis, fibrosis or calcification
4) adjacent focal muscular atrophy.^[9,10]^ In these aspects, a retrospective review of preoperative MRI findings from our patient shows iso- to slight hyper-intensity on T1-WI, iso-intensity on T2-WI, and homogenous enhancement of the entire lobulated mass on contrast-enhanced T1-WI with intra- and peri-tumoral signal voids. We think that lower signal intensities on T1- and T2-WI compared to the typical MRI features of IH may be associated with subacute hemorrhage inside the mass. However, preoperative accurate diagnosis of IH with hemorrhagic transformation is difficult considering the low incidence of IH itself and accompanying hemorrhagic change.

Hemangiomas can be subdivided into capillary, cavernous, and mixed-type, depending on whether capillary and cavernous predominant.^[7]^ Capillary hemangiomas account for 50% of all IH and 68% of head and neck IH. Capillary hemangiomas grow faster than the other types, with a high recurrence rate of around 20%. Cavernous hemangiomas consist of a large number of thin-walled vascular channels lined by a single layer of endothelial cells, which are separated by fibrous connective tissue, whereas capillary hemangiomas have no fibrous septa and smaller vascular lumens.^[11,12]^ Cavernous hemangiomas account for 26% of the head and neck IH with a recurrence rate of about 9%. Mixed-type hemangiomas account for only 5% of IH in the head and neck but have the highest recurrence rate of 28%.^[13]^ The IH of our case was identified as a mixed-type by histological examination, requiring a sufficient follow-up period for recurrence.

IH generally does not show natural regression and malignant change, but increased size and development of symptoms such as pain require surgical treatment. Local recurrence rate was reported at approximately 9% to 28% after surgical resection due to the infiltrative growth pattern.^[14]^ The recurrence may be related to the microscopically infiltrative pattern into the surrounding muscular tissue and minor arterial feeder vessels.^[15]^ Surgical margin and tumor size have been identified as independent factors predicting local recurrence, and incomplete resection can lead to an increasing rate of recurrence.^[16]^ The optimal treatment is complete resection including the muscles surrounding the tumor and controlling the feeding vessels to prevent recurrence. In a large-sized IH, preoperative angiography can be helpful to reduce intraoperative bleeding by the embolization of feeding arteries.^[17]^ In our patient, the tumor was removed completely including some of the surrounding muscles using the en bloc method with ligation of peritumoral vessels. MRI conducted after the 15-month follow-up period revealed no residual or recurrence lesion

While the pathophysiology of hemangiomas is not clearly defined, it is believed that abnormal angiogenesis may be involved.^[18]^ These processes are prompted by an increase in angiogenic factors such as vascular endothelial growth factors (VEGF) and matrix metalloproteinases (MMPs) and a decrease in anti-angiogenic factors.^[19,20]^ Tumor growth is promoted by high blood estrogen levels during puberty, pregnancy, oral contraceptive use, and androgen treatment.^[21]^ Hemorrhagic transformation of a hemangioma is rare. There are some cases involving hemorrhage in hepatic and vertebral hemangiomas have been documented in the literature, but to date, none have been reported in IH. In the cases of hepatic hemangioma, voluntary rupture occurs in 1% to 4%. Most of them are giant hepatic hemangioma (6–25 cm) with a mortality rate of 75%.^[22]^ In our case, the size of the hemangioma was relatively small and there were no specific factors related with tumor growth.

## 4 Conclusion

IH in the head and neck is uncommon, and hemorrhagic transformation is extremely rare. In the case of intramuscular tumors accompanied by a sudden onset of severe acute pain, we recommend considering a differential diagnosis of IH with hemorrhagic transformation. Complete resection of the tumor mass including surrounding muscles is required to prevent a recurrence.

## Author contributions

**Conceptualization:** Ikchan Jeon

**Data curation:** Ikchan Jeon

**Funding acquisition:** Ikchan Jeon

**Methodology:** Dongwoo Yu, Ikchan Jeon, Joon Hyuk Choi

**Resources:** Dongwoo Yu, Ikchan Jeon, Joon Hyuk Choi

**Supervision:** Ikchan Jeon

**Writing – original draft:** Dongwoo Yu

**Writing – review & editing:** Ikchan Jeon
